# Additional rare variant analysis in Parkinson’s disease cases with and without known pathogenic mutations: evidence for oligogenic inheritance

**DOI:** 10.1093/hmg/ddw348

**Published:** 2016-10-18

**Authors:** Steven J. Lubbe, Valentina Escott-Price, J. Raphael Gibbs, Mike A. Nalls, Jose Bras, T. Ryan Price, Aude Nicolas, Iris E. Jansen, Kin Y. Mok, Alan M. Pittman, James E. Tomkins, Patrick A. Lewis, Alastair J. Noyce, Suzanne Lesage, Manu Sharma, Elena R. Schiff, Adam P. Levine, Alexis Brice, Thomas Gasser, John Hardy, Peter Heutink, Nicholas W. Wood, Andrew B. Singleton, Nigel M. Williams, Huw R. Morris, for International Parkinson’s Disease Genomics Consortium

**Affiliations:** 1 Department of Clinical Neuroscience, Institute of Neurology, University College London, London, UK; 2 Department of Psychological Medicine and Neurology, Institute of Psychological Medicine and Clinical Neurosciences, Medical Research Council Centre for Neuropsychiatric Genetics and Genomics, Cardiff University School of Medicine, Cardiff, UK; 3 Laboratory of Neurogenetics, National Institute on Aging, Bethesda, MD, USA; 4 Department of Molecular Neuroscience, Institute of Neurology, University College London, London, UK; 5 Department of Clinical Genetics, VU University Medical Center (VUmc), Amsterdam, The Netherlands; 6 German Center for Neurodegenerative Diseases (DZNE), Tübingen, Germany; 7 Division of Life Science, Hong Kong University of Science and Technology, Hong Kong SAR, People’s Republic of China; 8 School of Pharmacy, University of Reading, Whiteknights, Reading, UK; 9 Centre for Integrated Neuroscience and Neurodynamics, University of Reading, Whiteknights, Reading, UK; 10 Reta Lila Weston Institute, University College London Institute of Neurology, Queen Square, London, UK; 11 Inserm U 1127, CNRS UMR 7225, Sorbonne Universités, UPMC Univ Paris 06 UMR S 1127, Institut du Cerveau et de la Moelle épinière, ICM, Paris, France; 12 Department of Neurodegenerative Diseases, Hertie Institute for Clinical Brain Research, Tübingen, Germany; 13 Centre for Genetic Epidemiology, Institute for Clinical Epidemiology and Applied Biometry, University of Tübingen, Tübingen, Germany; 14 Division of Medicine, University College London, London, UK and 15 UCL Genetics Institute, Department of Genetics, Environment and Evolution, University College London, London, UK

## Abstract

Oligogenic inheritance implies a role for several genetic factors in disease etiology. We studied oligogenic inheritance in Parkinson’s (PD) by assessing the potential burden of additional rare variants in established Mendelian genes and/or *GBA*, in individuals with and without a primary pathogenic genetic cause in two large independent cohorts totaling 7,900 PD cases and 6,166 controls. An excess (≥30%) of cases with a recognised primary genetic cause had ≥1 additional rare variants in Mendelian PD genes, as compared with no known mutation PD cases (17%) and unaffected controls (16%), supporting our hypothesis. Carriers of additional Mendelian gene variants have younger ages at onset (AAO). The effect of additional Mendelian variants in *LRRK2* G2019S mutation carriers, of which *ATP13A2* variation is particularly common, may account for some of the variation in penetrance. About 10% of No Known Mutation-PD cases harbour a rare *GBA* variant compared to known pathogenic mutation PD cases (8%) and controls (5%), with carriers having earlier AAOs. Together, the data suggest that the oligogenic inheritance of rare Mendelian variants may be important in patient with a primary pathogenic cause, whereas *GBA* increases risk across all forms of PD. This study highlights the potential genetic complexity of Mendelian PD. The identification of potential modifying variants provides new insights into disease mechanisms by potentially separating relevant from benign variants and by the interaction between genes in specific pathways. In the future this may be relevant to genetic testing and counselling of patients with PD and their families.

## Introduction

Significant progress has been made in identifying genes linked to Parkinson’s (PD). Mutations in several genes cause autosomal recessive or dominant Mendelian PD (1) and common variation in 28 variants across 24 loci increase PD risk (2). However, 5–10% of patients with PD, depending on the population, have a recognized pathogenic Mendelian cause, and common variants only contribute <5% of the genetic heritability (3), indicating that the majority of genetic inheritance is unexplained. Polygenic inheritance of common variants has been shown to be associated with early-onset PD (4). There is also evidence that multiple rare variants in Mendelian genes may increase the risk of some diseases in a non-Mendelian fashion in amyotrophic lateral sclerosis (5) and schizophrenia (6). Here, we define Known Mutation-PD as PD cases carrying a recognised high penetrance pathogenic mutation regardless of family history. Using genotype and exome sequence data in two large independent case-control cohorts, we therefore assessed the role of additional rare variants in PD genes and *GBA*, in patients with PD with and without a known genetic cause to determine if the inheritance of multiple variants might contribute to the development of PD.

## Results

A total of 181 rare variants within *GBA* and the established PD Mendelian genes on the NeuroX platform passed our QC criteria and were included in our analyses. Of these, 24 are known to be pathogenic (4 *LRRK2*, 19 *PARK2* and 1 *VPS35*), one variant is known to increase PD risk (1 *GBA*; rs76763715), with the remaining 156 variants having no known role in PD. From the exome data 228 rare variants were included in the analyses, and included 19 known pathogenic variants (2 *LRRK2*, 12 *PARK2*, 2 *PINK1*, 2 *SNCA* and 1 *VPS35*), three known risk variants (3 *GBA*), and 204 variants of unknown significance. PCA based on variants shared between the NeuroX and exome cohorts in relation to HapMap samples failed to reveal hidden population stratification.

In the NeuroX data, 1.3% (89/6,647) of PD samples had established PD-causing mutations (defining Known Mutation-PD cases), comprising single mutations in autosomal dominant genes (78 *LRRK2*, 1 *VPS35*) and biallelic mutations in autosomal recessive genes (10 *PARK2*). Similarly, 1.4% (18/1,253) of cases in the exome cohort harboured PD-causing mutations (9 *LRRK2*, 6 *PARK2*, 2 *SNCA* and 1 *VPS35*).

Focusing on the 107 Known Mutation-PD cases (Supplementary Material, Table S1), we investigated the potential increased burden of additional rare alleles in Mendelian PD genes separate to their primary causal mutation. We first looked at the proportion of cases carrying additional variants and observed that, after correction, statistically more NeuroX Known Mutation-PD cases (16.9%) carried additional variants compared to controls (9.2%; *P =* 0.019; *P*_corr _=_ _0.048; Table 1). This finding was replicated in our second dataset using exome data in which 33.3% of Known Mutation-PD exomes samples harboured additional variants compared to 15.4% of controls (*P =* 0.017). The role of additional Mendelian gene variants is corroborated by the increased burden of rare alleles in Known Mutation-PD cases as compared to controls in the NeuroX (OR = 1.70; 95% CI: 1.02–2.85; *P =* 0.043; Table 2). While this association did not withstand correction for multiple testing (*P*_corr _=_ _0.065), a similar pattern was also seen in the exome data (OR = 3.66; 95% CI: 1.15–11.68; *P =* 0.028).

**Table 1.** Number of Parkinson’s cases and controls in the NeuroX and exome cohorts harbouring additional rare variants in Parkinson’s Mendelian and risk genes

Number of additional variants	NeuroX (Cases = 6,647; Controls = 5,693)				Exome (Cases = 1,253; Controls = 473)			
	Mendelian		*GBA*		Mendelian		*GBA*	
	*N*	Freq	*N*	Freq	*N*	Freq	*N*	Freq
Known Mutation-PD^a^								
0	74	0.8315	82	0.9213	12	0.6667	18	1.0000
1	15	0.1685	7	0.0787	5	0.2778	0^c^	–
2	0	–	0	–	1	0.0556	0^c^	–
No Known Mutation-PD^b^								
0	5,881	0.8968	5,995	0.9142	1,031	0.8348	1,108	0.8972
1	634	0.0967	551	0.0840	181	0.1466	126	0.1020
2	38	0.0058	12	0.0018	20	0.0162	1	0.0008
3	5	0.0008	0		2	0.0016	0	–
4	0	–	0	–	1	0.0008	0	–
Controls								
0	5,169	0.9079	5,429	0.9536	400	0.8457	451	0.9535
1	499	0.0877	262	0.0460	69	0.1459	22	0.0465
2	24	0.0042	2	0.0004	4	0.0085	0	–
3	1	0.0002	0	–				

Abbreviations: Freq, frequency; *GBA*, Glucocerebrosidase; N, number of samples; PD, Parkinson’s disease.

a Samples with a known genetic cause identified.

b Samples without a known genetic cause.

c No carriers of risk alleles observed in the known mutation-PD samples.

**Table 2.** Investigation of additional rare alleles in known Parkinson's Mendelian and risk genes in the NeuroX and exome cohorts

	*N*	Mendelian genes		*GBA*	
		OR (95% CI)	*P*	OR (95% CI)	*P*
NeuroX					
Known Mutation-PD^a^ *vs*. Controls	89/5,693	1.70 (1.02–2.85)	0.043	1.88 (0.86–4.10)	0.112
No Known Mutation-PD^b^ *vs*. Controls	6,558/5,693	1.14 (1.02–1.28)	0.017	1.96 (1.69–2.28)	8.00E–19
Known Mutation-PD *vs*. No Known Mutation-PD	89/6,558	1.49 (0.90–2.46)	0.125	0.94 (0.44–2.04)	0.881
Exome					
Known Mutation-PD^a^ *vs*. Controls	18/473	3.66 (1.15–11.68)	0.028	0^c^	–
No Known Mutation-PD^b^ *vs*. Controls	1,235/473	1.05 (0.80–1.40)	0.711	2.39 (1.47–3.90)	0.0005
Known Mutation-PD *vs*. No Known Mutation-PD	18/1,235	1.51 (0.65–3.51)	0.343	0^c^	–

Abbreviations: CI, confidence intervals; N, Number of samples per group; OR, odds ratio; PD, Parkinson’s disease; P, logistic regression P-value correcting for gender, capture (exome only), population differences and platform (combined analysis only).

a Samples with a known genetic cause identified.

b Samples without an identified genetic causal.

c No carriers of risk alleles observed in the Known Mutation-PD samples.

In the context of all PD cases, we then tested to see if this observed enrichment was specific to Known Mutation-PD cases only. The proportion of additional variant carriers in Known Mutation-PD cases was higher than in No Known Mutation-PD cases in both cohorts but neither were significantly different (*P =* 0.058 and 0.143, respectively; Table 1). Similarly, the observed enrichment of additional alleles in the NeuroX (OR = 1.49, 95% CI: 0.90–2.46) and exome cohorts (OR = 1.51, 95% CI: 0.65–3.51; Table 2) failed to reach significance.

We next examined whether the presence of additional variants influences disease by modifying age at onset (AAO; Table 3). AAO data were available for 74 (83.1%) and 15 (83.3%) NeuroX and exome Known Mutation-PD samples, and for 5,713 (87.1%) and 1,130 (91.5%) No Known Mutation-PD samples. Although Known Mutation-PD samples with additional variants in the NeuroX cohort appeared to have had younger AAOs compared to those without, of approximately six years, this did not reach significance (*P =* 0.339). Younger AAOs, by approximately four years, was also seen in the exome cohort and again, this was not significant (*P =* 0.072).

**Table 3.** Average age of onset of Parkinson’s cases that harbour additional variants in known Mendelian and risk genes in the NeuroX and exome cohorts

	NeuroX				Exome			
Genes	With	Without	Coeff (95% CI)	*P*	With	Without	Coeff (95% CI)	*P*
All PD								
Mendelian	61.4	61.3	0.06 (−0.98; 1.09)	0.916	41.8	43.3	−1.34 (−2.98; 0.30)	0.109
*GBA*	59.3	61.5	−2.10 (−3.25; −0.95)	0.0003	42.4	43.1	−0.31 (−2.28; 1.67)	0.762
Known Mutation-PD^a^								
Mendelian	56.8	62.7	−3.78 (−11.62; 4.06)	0.339	38.3	42.3	−16.54 (−35.09; 2.01)	0.072
*GBA*	64.6	61.6	−0.23 (−11.75; 11.29)	0.968	0^c^	0^c^	–	–
No Known Mutation-PD^b^								
Mendelian	61.5	61.3	0.16 (−0.89; 1.20)	0.767	41.9	43.3	−1.29 (−2.94; 0.37)	0.127
*GBA*	59.2	61.5	−2.16 (−3.31; −1.00)	0.0003	42.4	43.1	−0.31 (−2.29; 1.66)	0.755

For each group, differences in the average age at onset were assessed by comparing cases harbouring additional variants to those without.

Abbreviations: CI, confidence interval; Coeff, regression coefficient; *GBA*, Glucocerebrosidase; PD, Parkinson’s disease; P, Linear regression P-value correcting for population, gender and capture (exome only);.

a Samples with a known genetic cause identified.

b Samples without a known genetic cause.

c No carriers of risk alleles observed in the Known Mutation-PD samples.

As expected, *LRRK2* was the most commonly mutated Mendelian gene identified in this study. In the NeuroX cohort, compared to controls, the majority of additional variants harboured by G2019S-positive *LRRK2* cases in both cohorts were within recessive PD genes (NeuroX: 14.7% *vs*. 5.7%, *P =* 0.007, *P*_corr _=_ _0.007; Table 4). This was replicated in the exome but did not reach independent significance (28.6% *vs*. 8.9%, *P =* 0.137). Interestingly, additional rare *ATP13A2* variants appeared enriched in NeuroX *LRRK2*-positive cases (8.0% *vs*. 2.9%, *P =* 0.004, *P*_corr _=_ _0.007). This enrichment was also seen in the exome cohort (14.3% *vs*. 1.7%, *P =* 0.017). Power constraints may have limited our ability to detect associations in the exome cohort, but associations observed in the NeuroX withstood correction for multiple testing. When considering all observed variation in Mendelian PD genes, 8.5% of all *LRRK2*-positive cases compared to 2.4% of controls harboured additional variants in *ATP13A2* (*P =* 0.004). Taken together, the data have consistently indicated an over-representation of additional variants in *ATP13A2* in *LRRK2*-positive cases. Due to very small numbers, the effect of additional *ATP13A2* variants in G2019S-carriers on AAO was only assessed in the NeuroX data and no statistical difference was observed (*P =* 0.178). There were no AAO differences in *ATP13A2* variant carriers across all PD cases or any of the groups.

**Table 4.** Enrichment of rare additional variants within known recessive Parkinson’s disease (PD) genes in *LRRK2*-mutation positive PD cases

	NeuroX	Exome	All variants
Cases			
Total *LRRK2* mutation positive	78	9	87
Total G2019S positive	75	7	82
With additional rare variants	13 (17.3%)	2 (28.6%)	15 (18.3%)
In recessive PD genes	11 (14.7%)	2 (28.6%)	13 (15.9%)
In *ATP13A2*	6 (8.0%)	1 (14.3%)	7 (8.5%)
Controls			
In recessive PD genes	323 (5.7%)	42 (8.9%)	379 (6.2%)
In *ATP13A2*	163 (2.9%)	8 (1.7%)	146 (2.4%)

Abbreviations: *ATP13A2*, ATPase 13A2; G, glycine; *LRRK2*, Leucine-rich repeat kinase 2; PD, Parkinson’s disease; S, serine.

The *LRRK2* G2019S mutation is more common in familial and sporadic Ashkenazi Jewish (AJ) patients with PD, as compared to other North American/European populations. Although we did not have AJ ethnicity information for our PD cases, we re-evaluated the data using AJ control samples. We specifically evaluated the possibility that the *ATP13A2* variants identified might be relatively common in AJ individuals and that our results might relate to population stratification. We identified 12 different additional rare variants evenly distributed within six different PD genes (Supplementary Material, Table S1), including five different *ATP13A2* non-Kufor-Rakeb syndrome (KRS) variants, in our *LRRK2*-positive cohort. We first investigated these variants using minor allele frequency (MAF) data from 3,044 AJ non-inflammatory bowel disease control samples to compare observed allele frequencies (Supplementary Material, Table S2; IBD Exomes Portal, Cambridge, MA; http://ibd.broadinstitute.org; date last accessed June 2016). All but one (*ATP13A2* p.P1100L, rs201756175) of the rare variants enriched in our *LRRK2*-positive samples were either not observed or were observed at lower/similar frequencies in the AJ controls as compared to the ExAc database. Removal of p.P1100L (which was only seen in the NeuroX cohort, and was more common in AJ controls) did not affect the results. Known Mutation-PD NeuroX samples (14.6%) still had more additional rare variants compared to No Known Mutation-PD (10.2%) and controls (9.0%), and *ATP13A2* variants were still over-represented in *LRRK2* G2019S carriers affected by PD. Secondly, using AJ defined MAFs, we assessed the burden of rare variants using individual level data from 318 AJ inflammatory bowel disease patients unaffected by PD (Supplementary Material, Table S6). All variants enriched in the Known Mutation-PD cases were rare or not observed in the 318 AJ samples (Supplementary Material, Table S2). Although underpowered, a smaller proportion of carriers of rare variants across all PD genes was also seen in the AJ control samples (18.9%; 60/318) as compared to the Known Mutation-PD samples. As in the initial analysis, a similar excess of *ATP13A2* variants was seen in the *LRRK2* G2019S PD cases compared to AJ control samples (14.3% *vs*. 5.0%; 16/318). Overall, our analysis does not suggest that the observed effects were due to population stratification but further analysis with larger AJ control datasets is recommended.

While no *GBA* variants were seen in the Known Mutation-PD exome group, we observed a higher proportion of carriers of additional *GBA* variants in the Known Mutation-PD NeuroX group compared to controls (7.9% *vs*. 4.7%; *P =* 0.106). Significantly increased proportions were also seen for the No Known Mutation-PD NeuroX samples compared to controls (*P =* 1.63E-18; Table 1). This association was still significant after correction (*P*_corr _=_ _4.80E-18) and was replicated in the exome data (*P =* 0.0005) therefore indicating that this effect was not confined to Known Mutation-PD. Evidence for allelic enrichment was observed for both Known Mutation-PD and No Known Mutation-PD as compared to controls in both cohorts (Table 2) suggesting that *GBA* variants are important across all forms of PD. There was a strong effect of *GBA* on AAO with NeuroX No Known Mutation-PD carriers of *GBA* variants having significantly lower AAOs than those without (on average 2.3 years; *P =* 0.0003, *P*_corr _=_ _0.0005). A trend in the same direction was noted for the exome cohort (Table 3). Specific biallelic *GBA* mutations cause Gaucher’s disease (GD). No sample appeared to have *GBA*-related GD. Interestingly the majority of the *GBA* risk variants in the NeuroX Known Mutation-PD group were all heterozygous for GD-causing mutations (5/7; 71.4%) as opposed to No Known Mutation-PD cases (64.1%; 361/563) and controls (164/264; 62.2%). Despite small numbers, NeuroX Known Mutation-PD samples harbouring single GD-associated *GBA* mutations appeared to have an excess of additional variants across all Mendelian genes studied (42.9%) (Supplementary Material, Table S3) as well as enrichment of additional *ATP13A2* variants (14.3%) compared to No Known Mutation-PD cases (2.5%, *P =* 0.147) and controls (1.2%, *P =* 0.128) (Supplementary Material, Table S4).

## Discussion

Here, we show in two large independent cohorts that PD cases with an established Mendelian genetic cause have an excess of additional rare variants of currently unknown significance in PD genes as compared to No Known Mutation-PD and controls. Carriers of additional rare variants also have a lower AOO by as much as 6 years in NeuroX and 4 years in exome data. Although neither finding was statistically significant, likely due to small number constraints, this provides preliminary evidence for the modifying effect of variants in additional genes in Mendelian PD. Overall, 33.3% of Known Mutation-PD cases have additional rare variants within established PD-causing genes compared to controls (15.6%) and No Known Mutation-PD cases (16.5%). Taken together the data, generated on different platforms (NeuroX rare variant genotyping chip and exome sequencing) that detected different numbers of variants, consistently provides evidence of possible oligogenic inheritance in Known Mutation-PD. Differences in the same direction between Known Mutation-PD and No Known Mutation-PD cases in both the exome and NeuroX cohorts suggest that the burden of additional rare variants might have a more prominent role in carriers of known high-penetrant mutations. In contrast, *GBA* does not have a specific effect in Known Mutation-PD as both PD groups had a comparable increase in *GBA* variant frequency as compared to controls.

Several large-scale studies have reported that the *LRRK2* G2019S mutation has reduced penetrance as well as phenotypic and/or pathological heterogeneity. Any modifying factors, aside from age, are unknown (7), and we infer that other genetic or environmental factors may influence *LRRK2*-disease. We found an enrichment of additional rare variants in recessive PD genes in *LRRK2*-cases in both cohorts and upon combining datasets. When considering all additional variants for both cohorts together, we identified that 6.2% of controls harbour an additional variant in a recessive PD gene, compared to 15.9% of *LRRK2*-positive Known Mutation-PD cases. Although underpowered, together the data consistently suggest that the presence of additional rare variants of unknown significance within recessive PD genes may influence penetrance. Typically, *LRRK2* mutation testing is offered to familial dominant cases or those from high-risk populations (8). Extending screening of *LRRK2*-positive cases to all known Mendelian PD genes as routine may uncover potential oligogenic variants and assist in elucidating the evident disease heterogeneity. Assessing potential oligogenic inheritance in a larger number of clinically unaffected *LRRK2* PD-mutation carriers would provide greater insight into the roles of additional variants in PD.

Lysosomal dysfunctional in PD is well established, with several PD genes having a primary lysosomal function (9). Enlarged lysosomes are a common phenotype in *LRRK2*-positive PD, as is increased lysosomal ATP13A2 expression (10). *ATP13A2* mutations cause KRS (MIM:606693). Additional variants enriched in G2019S-positive cases appear to cluster towards the C-terminus of isoform 3 of *ATP13A2* (NM_001141974) (Supplementary Material, Fig. S1). This has implications for cellular assessments in that studies on this gene in PD may need to be extended to all isoforms. While not causal for KRS, these variants may affect ATP13A2 function and modify existing disease in G2019S carriers. A similar enrichment of additional *ATP13A2* variants in *GBA* variant carriers was also observed. While the overlap of *GBA* and *ATP13A2* remains to be elucidated, our identification of a specific overlap between *GBA, ATP13A2* and *LRRK2* points to a convergence of *ATP13A2* and *LRRK2* in lysosomal function in PD.

A range of genetic variants appear to modulate AAO. We have previously described a role of both genome-wide significant and polygenic common variants in modulating PD AAO (4). In this work, we show that there is a reduced AAO in carriers of *GBA* mutations in PD cases in both cohorts and upon combining data sets together. We now have clear evidence that rare *GBA* risk variants and common GWAS-identified variants influence disease AAO. Further studies in larger datasets are needed to investigate the effect of rare variants in Mendelian genes and to fully explore the interplay between rare and common variants.

Population stratification is a significant concern, particularly when looking at individuals with a common founder mutation such as *LRRK2* G2019S. Together, the observed diversity of *ATP13A2* variants, the observed rarity of the additional variants observed in the *LRRK2*-positive samples within two AJ cohorts and, as in the initial analysis, the similar magnitude of excess of *ATP13A2* variants in *LRRK2* G2019S cases as compared to AJ controls, suggest that our results are not due to population stratification. However, further studies in large well-defined AJ population cohorts and in *LRRK2* G2019S families with variable penetrance are needed.

The results here are likely to be conservative estimates of oligogenic inheritance in PD. We have not comprehensively studied all incident Known Mutation-PD cases, not all pathogenic variants were included on the NeuroX platform, and the identification of Mendelian copy number variants (CNVs) was limited. The exome may have captured many more singletons which may be driving the observed results, while fewer represented variants on the NeuroX platform may have contributed to the observed power constraints. Additional studies are needed to help elucidate the role of additional PD gene variants in disease aetiology. The NeuroX CNV carrier frequency is lower than other studies (11), and this will have attenuated case-control differences. The variants were not confirmed by Sanger sequencing; however, any systematic bias in each cohort should be present for all samples. Similarly, not all CNVs were validated using multiplex ligation-dependent probe amplification (MLPA); however, all 13 samples that were available had the PennCNV-identified CNVs successfully validated.

In conclusion, we demonstrate that about 30% of Known Mutation-PD cases carry additional rare variants in PD genes compared to No Known Mutation-PD cases (16%) and controls (15%), and that carriers have a lower AAO. Although based on a large number of samples, only a small proportion carried primary pathogenic mutations and so our study is underpowered. Our findings, which were consistent across both cohorts provide evidence that the oligogenic inheritance of variants may be important in the development of PD. By acting as potential disease modifiers, additional rare variants in Mendelian genes, particularly *ATP13A2*, may account for some of the observed AAO heterogeneity in *LRRK2*-positive cases. As previously described, rare *GBA* variants are an important risk factor across all forms of PD. Further interpretation of oligogenic inheritance particularly in families with apparent reduced penetrance is needed. Although not all rare variants will have a biological effect, this study annotates a new set of variants that may influence disease development. The underlying reasons for the observed excess of additional variants in Known Mutation-PD cases as opposed to No Known Mutation-PD cases, where additional variants would be expected to also influence AAO, is unclear. This distinction between Known Mutation-PD and No Known Mutation-PD suggest that selective cellular pathways may be important in individuals with Known Mutation/Mendelian PD. Further work into the functional effect of these variants in model systems is needed. The clustering of modifying genetic factors in specific gene families in PD subgroups may provide further insights into pathways important in PD. In the future, genetic counselling in PD families may incorporate the potential disease modifying role of additional variants in order to improve the advice given to at-risk individuals.

## Methods and Materials

We first assessed high-quality genotype data derived from the NeuroX chip on 6,647 PD cases and 5,693 controls from the International Parkinson’s Disease Genomics Consortium (IPDGC; dbGaP Study Accession number: phs000918.v1.p1). Sample collection and variant genotyping are described elsewhere (12). We then used exome sequencing data on 1,253 independent PD cases and 473 controls, also from the IPDGC, as a replication data set. These cohorts were assembled for the discovery of new disease genes, and in some cases, samples with known disease genes were not submitted for analysis. The exome sequence samples were primarily derived from patients with early-onset PD.

Using annotated MAF data from 1000 Genomes Project (http://www.1000genomes.org/; date last accessed April 2015) and NHLBI GO Exome Sequencing Project (https://evs.gs.washington.edu/EVS/; date last accessed April 2015), all rare (MAF < 1%) variants (excluding synonymous) within ten known Mendelian PD genes and *GBA* (Supplementary Material, Table S5) were extracted and assessed from NeuroX and exome data. Variants and samples with >5% missing calls were excluded. Population outliers, based on principal components analysis (PCA) using HapMap population data, were also removed. Rare CNVs within *PARK2* were called from the NeuroX data using PennCNV (13). All CNVs spanned a minimum of 10 variants, were then visually confirmed and validated using MLPA where possible.

We specifically assessed the possibility of oligogenic inheritance in the pathogenesis of PD. This was done by investigating whether PD cases who harboured known primary Mendelian mutations, *i.e* well-established pathogenic dominant allele(s) or biallelic recessive alleles/CNVs (Known Mutation-PD cases), carried an excess of additional variants within (i) established Mendelian PD genes and (ii) *GBA*, compared to PD cases without a known Mendelian cause (which we refer to as No Known Mutation-PD) and unaffected controls. Pathogenic mutations were defined as those established mutations stringently defined as causing PD within the literature according to OMIM (http://omim.org/; date last accessed June 2016) and/or the Parkinson Disease Mutation Database (http://www.molgen.vib-ua.be/PDMutDB/; date last accessed June 2016).

Logistic regression was used to examine differences in the proportion of carriers of additional variants in Mendelian genes or *GBA* between Known Mutation-PD cases, No Known Mutation-PD cases, and unaffected controls. To assess allelic enrichment between groups, we summed the additional alleles per individual and remodeled the logistic regression. Within each group (Known Mutation-PD and No Known Mutation-PD), we compared carriers of additional variants to those without using linear regression to investigate the impact of additional variants on AAO. Fisher’s exact test was used to test differences in the number of *LRRK2*-positive PD cases with additional variants, when considering all observed variation, in both cohorts combined. To improve the power to detect enrichment, we also combined datasets while limiting these analyses to variants common to both platforms. The following covariates were used in all the above analyses: gender, capture metrics (exome only), PCA components (1–4) and platform (for combined analyses only). To account for multiple testing issues we applied a false discovery rate (FDR) threshold <0.05 (14).

## Supplementary Material

Supplementary Material is available at *HMG* online.

## Supplementary Material

Supplementary Data
